# A Physical Activity Intervention and Changes in Body Mass Index at a Middle School With a Large American Indian Population, Oklahoma, 2004–2009

**DOI:** 10.5888/pcd13.150495

**Published:** 2016-12-01

**Authors:** June E. Eichner, Olakunle A. Folorunso, William E. Moore

**Affiliations:** 1 University of Oklahoma Health Sciences Center, Oklahoma City, Oklahoma.

## Abstract

School-based interventions can reach children and adolescents and aid in reducing the prevalence of childhood obesity. A physical education class that engaged middle school students in a daily 1-mile walk or run and other team sports was developed in a rural school in southwestern Oklahoma with a large American Indian population. Body mass index *z* scores decreased among boys and were stable among girls in the intervention group compared with students who did not participate in the intervention. A daily required walk or run may help to establish a physical activity habit with all of its associated benefits.

## Objective

Even with the recent reports of stabilizing rates, the prevalence of childhood overweight and obesity is at an all-time high (1). American Indian children have disproportionately high rates of overweight and obesity (2,3), but because these populations of children are small and isolated in rural areas, intervention studies rarely include them. The objective of this study was to compare changes in body mass index (BMI) *z* scores of participants enrolled in a physical activity intervention at a middle school in southwestern Oklahoma with the BMI z scores of the nonparticipating school population.

## Methods

The Anadarko Public School System, with approximately 2,000 students in grades kindergarten through 12, serves a largely American Indian population from various tribes, including Apache, Fort Sill Apache, Caddo, Comanche, Delaware, Kiowa and Wichita. Sixty-five percent of the students are American Indian, 25% are white, 5% are African American, and 5% are Latino. The poverty rate in the district population is about twice the state average, the unemployment rate is 3 times higher, and the average household income is 82% of the state average (4). Study participants were sixth-, seventh-, and eighth-grade students aged 12 to 15 years attending Anadarko Middle School, which has an average fall enrollment of 442 (4). 

We developed the program, Middle School Opportunity for Vigorous Exercise (MOVE), with assistance from the school’s physical education teacher and principal. The program was based on an exercise prescription suggested by the 1996 Report of the Surgeon General, *Physical Activity and Health* (5). The program’s objective was to habituate middle school students to daily walking or running. Participants walked or ran 1 mile each school day and then engaged in a team activity such as basketball, soccer, football, dodge ball, or volleyball. Class size was approximately 20 students. The intervention took place during 5 school years beginning in August 2004 and ending in May 2009.

We measured changes in BMI among participants and nonparticipants. To be included in this analysis, data had to be for first-time MOVE participants who completed 2 consecutive semesters of the class (fall and spring of 1 school year) and had both pre-intervention and post-intervention BMI measurements (weight [kg]/height [m^2^]). Some students attended the MOVE class for only 1 semester or several nonconsecutive semesters. At the study’s midpoint (2006–2007), we measured height and weight of all available middle school students at the beginning and end of the school year. We measured changes from pre-intervention to post-intervention in BMI *z* score and BMI percentile according to the 2000 Centers for Disease Control and Prevention (CDC) Growth Charts (6). We analyzed differences in changes between the intervention and comparison groups using repeated measures analyses of variance (ANOVA) and Statistica software (StatSoft [www.statsoft.com]); an α of less than .05 determined significance. Approval was obtained from the University of Oklahoma Health Sciences Center institutional review board.

## Results

During the study period, 182 unique students enrolled in the MOVE class; of these, 50 students transferred out of the school or district or joined a sports group or band. Sixty-six students (46 boys, 20 girls) met the criterion of 2-semester consecutive attendance in the MOVE class (Table 1). Of these, 57.6% were American Indian; 19.7% were white, 15.2% were Latino, 6.1% were African American, and 1.5% were Asian. At pre-intervention assessment, 10 of 66 (15.2%) students were overweight, and 19 (28.8%) were obese. Mean BMI *z* scores remained the same among girls participating in MOVE (from 0.7 to 0.7) and increased for nonparticipating girls (from 1.1 to 1.2). Mean BMI *z* score decreased among boys participating in MOVE (from 0.8 to 0.7) and increased among nonparticipating boys (from 1.1 to 1.2).

**Table 1:** Characteristics at Pre-Intervention Assessment of Participants in MOVE Physical Education Class and Nonparticipants, Anadarko, Oklahoma, 2004–2009

Variable	Nonparticipants (n = 287)	MOVE Participants (n = 66)	*P* Value
**Age (SD), y**	12.8 (1.0)	13.4 (0.8)	<.01^a^
**Sex, n (%)**			
Female	158 (55.1)	20 (30.3)	<.01^b^
Male	129 (44.9)	46 (69.7)	
**Race, n (%)**			
American Indian	178 (62.0)	38 (57.6)	.58^b^
Other	109 (38.0)	28 (42.4)	
**BMI status, n (%)**			
Underweight	4 (1.4)	2 (3.0)	.23^b^
Healthy weight	121 (42.2)	35 (53.0)	
Overweight	60 (20.9)	10 (15.2)	
Obese	102 (35.5)	19 (28.8)	
**BMI percentile mean (SD)**	78.6 (24.7)	69.6 (31.1)	.01^a^
**BMI *z* score^c^ mean (SD)**	1.1 (1.0)	0.8 (1.3)	.03^a^
**BMI *z* score^c^ mean converted to percentile (SD)**	86.9 (84.6)	77.6 (89.6)	—

Abbreviations: BMI, body mass index; MOVE, Middle School Opportunity for Vigorous Exercise.

a Determined by *t* test.

b Determined by Fisher exact test.

c BMI *z* score is based on Centers for Disease Control and Prevention 2000 growth charts (5).

MOVE participants overall (boys and girls) had a significantly smaller increase in BMI *z* score than did nonparticipants (BMI *z* score × treatment, *P* = .01) (Table 2). We found no significant difference between sexes when we compared changes in BMI z scores of participants with those of nonparticipants (BMI *z* score × treatment × sex; *P* = .49). 

**Table 2:** Repeated Measures Analysis of Variance of BMI *z* Score^a^: MOVE Participants vs Nonparticipants, Anadarko, Oklahoma, 2004–2009

Measure	Sum of Squares	Degrees of Freedom	Mean Square	*F*	*P* Value
**Between participants**					
Intercept	326.48	1	326.47	150.05	<.001
Sex	0.01	1	0.01	0.01	.93
Treatment	16.75	1	16.75	7.70	.01
Sex × Treatment	0.16	1	0.16	0.08	.78
Error	759.32	349	2.18	—	—
**Within participants**					
BMI *z* score	0.02	1	0.02	0.64	.42
BMI *z* score × sex	0.09	1	0.09	3.15	.08
BMI *z* score × treatment	0.18	1	0.18	6.62	.01
BMI *z* score × sex × treatment	0.01	1	0.01	0.49	.49
Error	9.52	349	0.03	—	—

Abbreviation: BMI, body mass index; MOVE, Middle School Opportunity for Vigorous Exercise.

a BMI *z* score is based on Centers for Disease Control and Prevention 2000 growth charts (5).

## Discussion

Overall, middle school students participating in the MOVE class for 1 year had better BMI *z* scores than nonparticipating students. Among MOVE participants, mean BMI *z* scores decreased for boys and were stable for girls. Among nonparticipants, mean BMI *z* scores increased for both sexes. The large number of students not completing the 1-year MOVE sequence was due to competing requirements, transfers out of the school system, and sports or band practice. MOVE was not a required course; students with the greatest need for the program may have dropped out. Although our program was small compared with large, multicenter trials, it was structured to achieve a predetermined amount of movement each day. Health education and a curriculum that encourages a healthy lifestyle are important, but a minimal amount of daily walking or running may also be required to maintain or decrease BMI. Our physical activity program showed evidence of impact on the primary outcome of BMI *z* score. Conducted under real-world circumstances, our program demonstrated that improvements in BMI can be achieved by providing a well-regimented program of moderate to vigorous physical activity.
